# High-Throughput Analysis of the T Cell Receptor Beta Chain Repertoire in PBMCs from Chronic Hepatitis B Patients with HBeAg Seroconversion

**DOI:** 10.1155/2016/8594107

**Published:** 2016-10-13

**Authors:** Yachao Qu, Yong Huang, Di Liu, Yinuo Huang, Zhiyi Zhang, Zhiqiang Mi, Xiaoping An, Yigang Tong, Jun Lu

**Affiliations:** ^1^Hepatology and Cancer Biotherapy Ward, Beijing You'an Hospital, Capital Medical University, Beijing, China; ^2^State Key Laboratory of Pathogen and Biosecurity, Beijing Institute of Microbiology and Epidemiology, Beijing, China; ^3^Network Information Center, Institute of Microbiology, Chinese Academy of Sciences, Beijing, China

## Abstract

T lymphocytes are the most important immune cells that affect both the development and treatment of hepatitis B. We used high-throughput sequencing to determine the diversity in the V and J regions of the TCR*β* chain in 4 chronic hepatitis B patients before and after HBeAg seroconversion. Here, we demonstrate that the 4 patients expressed Vβ12-4 at the highest frequencies of 10.6%, 9.2%, 17.5%, and 7.5%, and Vβ28 was the second most common, with frequencies of 7.8%, 6.7%, 5.3%, and 10.9%, respectively. No significant changes were observed following seroconversion. With regard to the J*β* gene, J*β*2-1 was the most commonly expressed in the 4 patients at frequencies of 5.8%, 6.5%, 11.3%, and 7.3%, respectively. Analysis of the V-J region genes revealed several differences, including significant increases in the expression levels of V7-2-01-J2-1, V12-4-J1-1, and V28-1-J1-5 and a decrease in that of V19-01-J2-3. These results illustrate the presence of biased TCRV*β* and J*β* gene expression in the chronic hepatitis B patients. TRBV*β*12-4, V*β*28, J*β*2-1, V7-2-01-J2-1, V12-4-J1-1, and V28-1-J1-5 may be associated with the development and treatment of CHB.

## 1. Introduction

According to the World Health Organization, two billion people worldwide are infected with hepatitis B virus (HBV), and 350 million people are chronically infected. Approximately one million people die each year due to HBV infection, which leads to cirrhosis, liver failure, and/or hepatocellular carcinoma [1]. Therefore, understanding the pathogenesis of chronic hepatitis B (CHB) is of great importance. The natural history of HBV is influenced by the host, virus, and microenvironment [2]. Circumstantial evidence suggests that HBV does not directly lead to hepatocyte necrosis. Hepatocyte injury caused by HBV is believed to be mediated by the cellular immune response against the virus [3]. This response is relatively strong in acute, self-limited HBV infection. However, circulating HBV-specific T cells are rarely detected in CHB [4].

A healthy adult has approximately 2.5 × 10^7^ different polyclonal T cells, each of which expresses a particular T cell receptor (TCR) [5]. TCR is a heterodimer consisting of *α* and *β* protein chains that determine T lymphocyte specificity [6]. TCR diversity is generated by the somatic recombination of the V, D (for TCR*β* only), and J gene segments [7]. In the process of T cell maturation, allelic exclusion ensures that only one *β* chain protein is expressed in T cells [8]. Therefore, TCR*β* chains can be used as molecular fingerprints to identify T cell clones. Human TCR*β* includes 48 different functional V, 13 J, and 2 D gene segments in addition to 1 J and 18 V pseudogenes, which can be rearranged [9]. A number of techniques have been applied to analyze the TCR repertoire [10]; for example, flow cytometry is commonly employed; however, this method is limited by anti-TRB antibody specificity [11]. Gene melting spectral pattern (GMSP) assay, which is used to analyze the TCR gene family, is widely used [12]. Because this technique requires a large number of PCR reactions including primers for all possible V and J genes, its application is limited. Delayed data availability and the inability to detect variations in TCR*β* chain sequences are further drawbacks. More powerful diagnostic tools are therefore needed to accurately assess TCR diversity that can be used to monitor immune reconstitution, therapeutic responses, and disease status and to identify the T cell clonotypes in different diseases.

In recent years, next-generation sequencing (NGS), a fast and accurate new method, has been applied in various fields of medicine, with high coverage and massively parallel DNA sequence identification [13, 14]. In this study, we collected peripheral blood mononuclear cells (PBMCs) from CHB patients undergoing treatment. Using NGS, we compared the TCR*β* repertoire before and after HBeAg seroconversion in these patients and determined the pathogenesis of CHB during treatment. This study has generated new ideas for the development of effective individual treatment strategies and research technology platforms.

The purpose of our study was to elucidate the molecular portrait of TCR*β* chains in PBMCs from CHB patients and to reveal the role of cell-mediated immunity in the pathogenesis of chronic HBV infection to facilitate the development of individualized treatment.

## 2. Materials and Methods

### 2.1. Subjects

Between April 2012 and July 2013, 18 patients with hepatitis B surface antigen (HBsAg) that tested positive for at least 6 months were enrolled in our study. The subjects were selected at the Beijing You'an Hospital, affiliated with Capital Medical University. Individuals with hepatitis C or D or human immunodeficiency (HIV) virus or autoimmune disease and other malignancies were excluded. This study was performed in accordance with the principles of the Declaration of Helsinki. All patients provided informed consent before the initiation of the study. Each patient was treated orally with 0.5 mg entecavir (Bristol-Myers Squibb, USA) once a day for 48 weeks and injection of 1.6 mg thymosin *α*1 (Patheon Italia SPA, Italy) every other day for 24 weeks. Every three months, the patients visited the outpatient department for examination.

### 2.2. Serological and Biochemical Assays

ALT and TBIL levels were measured with an automatic biochemical analyzer (Beckman 5400, California, USA), and HBV DNA was quantified using real-time fluorescence quantitative PCR (Applied Biosystems 5700, California, USA) according to the manufacturer's protocol. HBsAg, HBsAb, HBeAg, anti-Hbe, and anti-HBc levels were assessed by enzyme immunoassays (Roche E601, Basel, Switzerland).

### 2.3. Isolation and Cryopreservation of PBMCs

PBMCs were prepared from 2 mL of whole blood collected from CHB patients by Lymphoprep (Axis-Shield, Oslo, Norway). Approximately 1 × 10^6^ PBMCs can be obtained with this method. Cells were mixed into 1 mL RPMI 1640 medium (Life, New York, USA), which contains 10% MDSO and 20% bovine serum, and then placed in a Cryo 1° Freezing Container (Nalgene, USA) for storage at −80° until use in the experiments.

### 2.4. Total RNA Isolation and cDNA Synthesis

Total RNA was extracted from PBMCs using RNA (RNeasy kit) according to the manufacturer's instructions, and cDNA synthesis was performed immediately with cDNA (Superscript II).

### 2.5. PCR cDNA Amplification and TCR*β* Chain Sequencing

PCR was performed with a Thermal Cycler PCR System 2720 (Gene Company Limited). One round of PCR was carried out to amplify cDNA. Each 50 *μ*L PCR reaction contained 2 *μ*L cDNA, 12 *μ*L ddH_2_O, 25 *μ*L Premix Ex Taq (TaKaRa), 200 nM of the specific primer CP1 (GCACCTCCTTCCCATTCAC, which targets C region genes [15]), and 2 *μ*M of the degenerate primer VP1 (GCIITKTIYTGGTAYMGACA, which targets the V region and covers 42 V*β* chains [15]). The PCR program was as follows: one cycle at 94° for 10 min, followed by 40 cycles at 94° for 30 s, 50° for 30 s, and 72° for 30 s, with a final 10 min extension at 72° and a 4° hold. Ten milliliters of each PCR product was run on a 2% agarose gel (150 V, 45 min), and products of approximately 400 bp were excised and purified using a Gel Extraction Kit (Qiagen, Hilden, Germany). The library was prepared according to the Ion Torrent sequencing manufacturer's instructions. The PGM sensed the H+ signal as sequencing-by-synthesis progressed [16].

### 2.6. Analysis of TCR Diversity

A Blast+ [17] search was carried out for the alignment-based identification of individual amplicons against TCR*β* chain germ line genes (63 TRBV, 2 TRBD, and 14 TRBJ), which were derived from IMGT/GENE-DB database (http://imgt.cines.org/). A homemade Perl script was used to analyze the Blast results and quantify the TRBV and TRBJ gene pairings, including the TRBV-only and TRBJ-only amplicons.

## 3. Results

### 3.1. Clinical Outcomes

After antiviral treatment with entecavir and thymosin *α*1 for 48 weeks, 98% (17/18) of the patients had no detectable serum HBV DNA, the serum ALT level returned to normal, and 22.2% (4/18) of the patients achieved HBeAg seroconversion. No patient had undergone HBsAg seroconversion by week 48 of treatment. PBMCs were collected from the 4 patients before and after HBeAg seroconversion for TCR sequencing, and their clinical characteristics are summarized in Table 1.

*Sequencing Data*. The total numbers of sequencing reads for the 4 paired samples ranged from 370,210 to 685,596, with an average length of approximately 230 bp (Table 2). The average length may have been smaller than the library length because the PGM prevented some of the PCR amplicons from being fully sequenced. The ratio of Q20 (quality score of ≥20) bases to total bases sequenced ranged from 83.2% to 86.2%, indicating a high level of sequencing quality.

### 3.2. Biased V*β* and J*β* Gene Segments within TCRs

**Figure 1:**
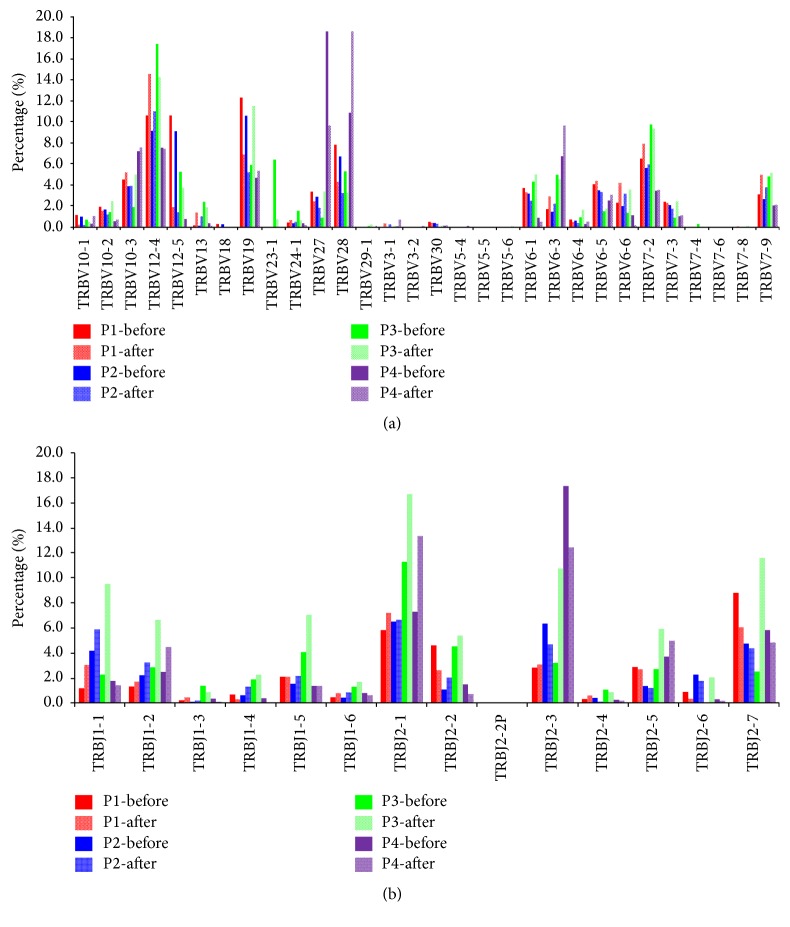
The serial results of relative TCRV*β* and J*β* expression in the four patients (P1–P4) before and after HBeAg seroconversion. (a) The serial results of relative TCRV*β* expression in the four patients (P1–P4) before and after HBeAg seroconversion. (b) The serial results of relative J*β* expression in the four patients (P1–P4) before and after HBeAg seroconversion.

Among the productively rearranged clones from the PBMCs of the patients, 30 V*β* gene segments and 14 J*β* gene segments were identified. The serial results of relative TCRV*β* and J*β* expression in the four patients (P1–P4) before and after HBeAg seroconversion are shown in Figure 1.

The gene fragments, which initially accounted for more than 5%, were defined as the advantage segments. The expression levels of the V*β* genes were diverse. V*β*12-4 was the most common segment detected in the PBMCs of the four patients, with frequencies of 10.6%, 9.2%, 17.5%, and 7.5%, respectively. V*β*28 was also frequently found in the four patients at frequencies of 7.8%, 6.7%, 5.3%, and 10.9%, respectively. V*β*12-5 (10.6%, 9.1%, and 5.3%), V*β*19 (12.3%, 10.6%, and 5.9%), and V*β*7-2 (6.5%, 5.6%, and 9.8%) were frequently observed in three of the patients, while other gene segments were detected in less than 5% of the patients. Furthermore, we analyzed the differences between the two periods (before and after HBeAg seroconversion). A greater than 3% fluctuation in the relative TCRV*β* gene expression ratio between the two stages was defined as a significant change. Several TCRV*β* families showed changes between the two time periods (P1: V*β*12-4 was upregulated and V*β*12-5, V*β*19, and V*β*28 were downregulated; P2: V*β*12-5, V*β*19, and V*β*28 were downregulated; P3: V*β*10-3 and V*β*19 were upregulated and V*β*12-4 and V*β*23-1 were downregulated; and P4: V*β*28 was upregulated and V*β*27 was downregulated). However, no common highly fluctuating TCRV*β* segment was found in the patients. Moreover, there was no correlation between the changes in TCRV*β* gene expression and the serum ALT or TBIL level.

With regard to J*β* gene expression, J*β*2-1 was the most abundant segment in the PBMCs of the four patients at frequencies of 5.8%, 6.5%, 11.3%, and 7.3%, respectively. J*β*2-3 (6.4% and 17.3%) and J*β*2-7 (8.8% and 5.8%) were frequently expressed in two of the patients, while other gene segments were detected in less than 5% of cells from most of the patients. We also analyzed the changes in TCRJ*β* gene expression between the two periods. There were no significant differences between the two stages for the first two patients (P1 and P2). However, several J*β* segments were upregulated in the third patient (P3) (J*β*1-1, J*β*1-2, J*β*1-5, J*β*2-1, J*β*2-3, J*β*2-5, and J*β*2-7), and J*β*2-1 was upregulated, while J*β*2-3 was downregulated, in the fourth patient (P4). Given that the third patient (P3) was the only female of the four subjects, it is possible that TCRJ*β* expression is affected by gender, further influencing the efficacy of therapy. Similarly, there was no correlation between the changes in TCRJ*β* gene expression and the serum ALT or TBIL level.

### 3.3. Restricted Changes in TCR*β*V-J Gene Pairing in the Four Patients

**Figure 2:**
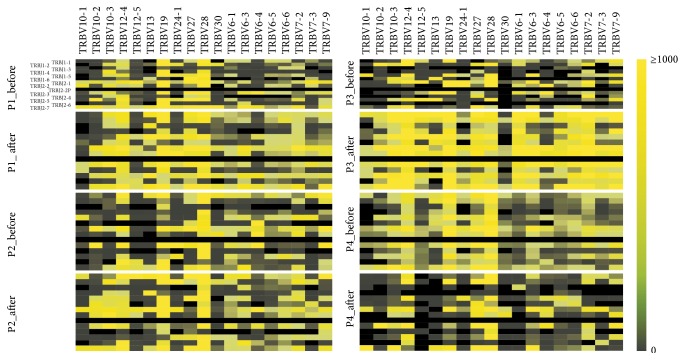
The serial results of TCR*β*V_J gene pairings in the four patients (P1–P4) before and after HBeAg seroconversion. TCR*β*V_J gene pairings were detected in the raw reads of four patients separately (both before and after treatment). Among the patients, there were several combinations that tended to be similarly differentially expressed following the shift from HBeAg positive to negative.

The sequencing reads showing similarity to certain V and J genes were defined as TCR*β*V_J gene pairings. TCR*β*V_J gene pairings were detected in the raw reads of four patients separately (both before and after treatment). Differences in the expression of each combination detected in the four patients are shown in Figure 2. Among the patients, there were several combinations that tended to be similarly differentially expressed following the shift from HBeAg positive to negative. For example, the combinations V7-2-01-J2-1, V12-4-J1-1, and V28-1-J1-5 were upregulated and V19-01-J2-3 was downregulated significantly following seroconversion.

## 4. Discussion

HBV infection is a major public health concern because it has significant impacts on human health. The different outcomes of HBV infection are determined by the immune status of the host. In acute self-limited HBV infection, the T cell response is strong, while it is relatively weak in patients with CHB [18]. Moreover, Dou et al. [19] have demonstrated that the population of HBcAg-activated T cells is altered during the course of CHB. The TCR*β* chain can reflect the status and role of T cells. There are also three complementarity-determining regions (CDR1, CDR2, and CDR3) in the TCR. Molecular structural analysis has revealed that CDR3 mainly recognizes MHC molecules bound to antigenic peptides [20, 21]. Hence, analysis of CDR3 can reveal changes in antigen-stimulated T cells [22, 23], the number of T cell clones, and T cell functioning [24, 25].

Analysis of TCR usage in patients can aid in the understanding of immune responses under a number of conditions during the course of a disease [26]. Sugyo et al. [27] monitored the TCR*β* chains of 4 healthy controls from 4 to 8 weeks, revealing that there was no significant change in the ratio. Many studies have confirmed that, in patients with viral infection or cancer, the frequency of antigen-specific TCRs differs [28, 29]. In our study, we observed several patients with the increased and/or decreased expression of TCR families, which is consistent with previous studies suggesting that the T cell response to HBV employs diverse TCR families [12, 30]. The findings of this study are also in agreement with those of prior studies demonstrating that TCR families are biased [31]. These observations may be correlated with the different epitopes of HBV or to the different HLA phenotypes [31, 32].

Most patients with CHB are successfully treated with antiviral drugs. However, some require long-term therapy, and relapse is common. At present, HBV therapy is primarily based on the uses of nucleoside analogs and immunomodulating agents. The effects of these treatments are closely correlated with the rescue of T cell function, especially among HBV-specific T cells [4]. Furthermore, these treatments are often limited by drug resistance and side effects. Moreover, the response rate is low, and many patients subsequently relapse [33]. Hence, it is necessary to develop new interventions for HBV infection.

In recent years, many researchers have modified T cells with specific TCR genes to cure cancer and other diseases. Morgan et al. [34] have demonstrated that TCR gene-modified T cells could be used for treating melanoma. Xue et al. [35] engineered patients' T cells to express WT1-TCR, which eliminates autologous leukemia progenitor cells, in an in vivo model. Their findings provide a strong basis for the planned WT1-TCR gene therapy trials of leukemia patients. Gehring et al. [36] used vector-mediated gene transfer to introduce HLA A2-restricted, HBV-specific TCRs into T cells of chronic HBV and HBV-related HCC patients and found that these genetically modified T cells could be used to reconstitute virus-specific T cell immunity in the chronic HBV patients and target tumors in the HBV-related HCC patients. In 2013, Koh et al. [37] performed electroporation of mRNA-encoding anti-HBV TCRs to explore a safer and more practical method for cell therapy of HCC that may also be employed to treat other HBV-related diseases. Krebs et al. [38] have demonstrated that T cells with a chimeric antigen receptor (CAR) specific for HBV envelope proteins localized to the livers of mice to reduce HBV replication cause only temporary damage. This immune therapy could be developed for CHB patients, regardless of their HLA type. Similar studies have also been conducted on acute hepatitis B and chronic severe hepatitis B patients [39, 40].

NGS was carried out to analyze TCR expression before and after HBeAg seroconversion in patients with CHB. High-throughput sequencing has been used to monitor the drug resistance of HBV [41]. Recently, Robins et al. [42] have studied TCR diversity in 2 healthy adults based on NGS and spectratyping, similarly showing the improved sensitivity of the former. Han et al. [43] have used high-throughput sequencing and suggested that comparison of the T cell repertoires of tissue and blood could be used to distinguish liver cancer patients from healthy adults and from hepatitis patients. Moreover, recent study of Huang et al. [44] has demonstrated that the genomic rearrangement of the V and J segments of TCR*β* chain V area may be associated with the chronic progression of HBV and impact on treatment efficacy. We used this method to determine the exact length and sequence of CDR3 to identify TCRV*β* and J*β* genes.

A comparison of the two regions indicated that the TCR*β* families V*β*12-4, V*β*12-5, V*β*19, V*β*28, V*β*7-2, J*β*2-1, J*β*2-3, and J*β*2-7 were more frequently expressed in the HBeAg-positive patients than the other TCR family members. These results are consistent with those of other studies suggesting that TCRV*β*7 and V*β*12 are more abundant than other TCRV*β* genes in HBV-related infections [19, 40, 45] and other diseases [46].

Moreover, many V*β*-J*β* gene segment combinations were found to change with HBeAg status, and this activity may be specific to HBV antigens. However, it was difficult to identify the HBV peptides that these TCRs reacted to. Such biased usage of TCRs has also been reported by studies of multiple sclerosis, primary biliary cirrhosis, and autoimmune hepatitis [47–51]. Still, the identification of TCR families can help to elucidate the pathogenesis of and improve the treatment of hepatitis B.

In conclusion, the characteristics of TCRs in CHB patients are biased and involve multiple TCR*β* families. The TCR*β*V7-2-01-J2-1, V12-4-J1-1, V28-1-J1-5, and V19-01-J2-3 genes may contribute more to the emergence and maintenance of anti-HBe in CHB patients and may represent potential targets for a therapeutic vaccine for CHB. Crystal structure studies are needed in the future.

## Supplementary Material

We have also do some research on T cell functional assays. Compared with the baseline, the percentage of Cytotoxic T lymphocytes(CTL) has been increased after HBeAg seroconversion which demonstrated the CTL play an important role in the treatment of HBV. The results of percentage of CTL in the patient 2 and 3 before and after HBeAg seroconversion are shown in Supplement data.

## Figures and Tables

**Table 1:** Clinical characteristics of the enrolled patients.

Patient	Gender	Age (year)	ALT (IU/L)		TBIL (*μ*mol/L)		HBV DNA (IU/mL)		Test for hepatitis B		HBsAg (IU/mL)	
			Before	After	Before	After	Before	After	Before	After	Before	After
P1	M	25	560.1	35.6	27.2	12.2	1.64 × 10^7^	<100	135+	145+	2909	296.6
P2	M	22	149.9	33	15.3	12.5	6.26 × 10^3^	<100	135+	145+	5505	522.4
P3	F	19	331.4	39.6	5.3	9.9	2.60 × 10^4^	<100	135+	145+	49394	227.6
P4	M	39	60	37.3	14.3	14.9	2.21 × 10^5^	<100	135+	145+	9273	1968

M: male; F: female; ALT: alanine aminotransferase; TBIL: total bilirubin; 135+: HBsAg, HBeAg, and anti-HBc positive; 145+: HBsAg, anti-Hbe, and anti-HBc positive.

Normal values: ALT ≤ 40 IU/L; TBIL ≤ 21 *μ*mol/L.

**Table 2:** The number of raw sequencing reads and the rearranged TRBV_J clones.

Samples	Raw reads^*∗*^	Average length (bp)	TRBV_J gene pairing	TRBV-only gene	TRBJ-only gene	Nonhomologous
P1_pre	526,527	256	122,007	256,443	33,484	69,906
P1_post	526,527	224	97,239	263,323	60,92	87,755
P2_pre	685,596	233	119,794	252,164	59,132	129,358
P2_post	685,596	228	150,460	332,818	83,253	106,459
P3_pre	607,994	216	169,465	228,845	79,726	96,378
P3_post	607,994	236	181,292	231,489	73,901	82,747
P4_pre	370,210	242	101,415	181,392	39,651	37,718
P4_post	370,210	232	251,846	37,457	35,291	27,889

^*∗*^The reads used for analyses were adjusted from the raw sequencing data to guarantee that each patient has the same sample size in both pre- and posttreatments.

TRBV: TCR beta chain variable gene; TRBJ: TCR beta chain junction gene.
