# Supplementary material for: High-Throughput Analysis of the T Cell Receptor Beta Chain Repertoire in PBMCs from Chronic Hepatitis B Patients with HBeAg Seroconversion
Source: Can J Infect Dis Med Microbiol. 2016 Oct 13;2016:8594107. doi: 10.1155/2016/8594107 (PMC5081459; doi:10.1155/2016/8594107)
Supplement: Supplementary file 1 — We have also do some research on T cell functional assays. Compared with the baseline, the percentage of Cytotoxic T lymphocytes(CTL) has been increased after HBeAg seroconversion which demonstrated the CTL play an important role in the treatment of HBV. The results of percentage of CTL in the patient 2 and 3 before and after HBeAg seroconversion are shown in Supplement data. [file 8594107.f1.ppt]

## Slide 1
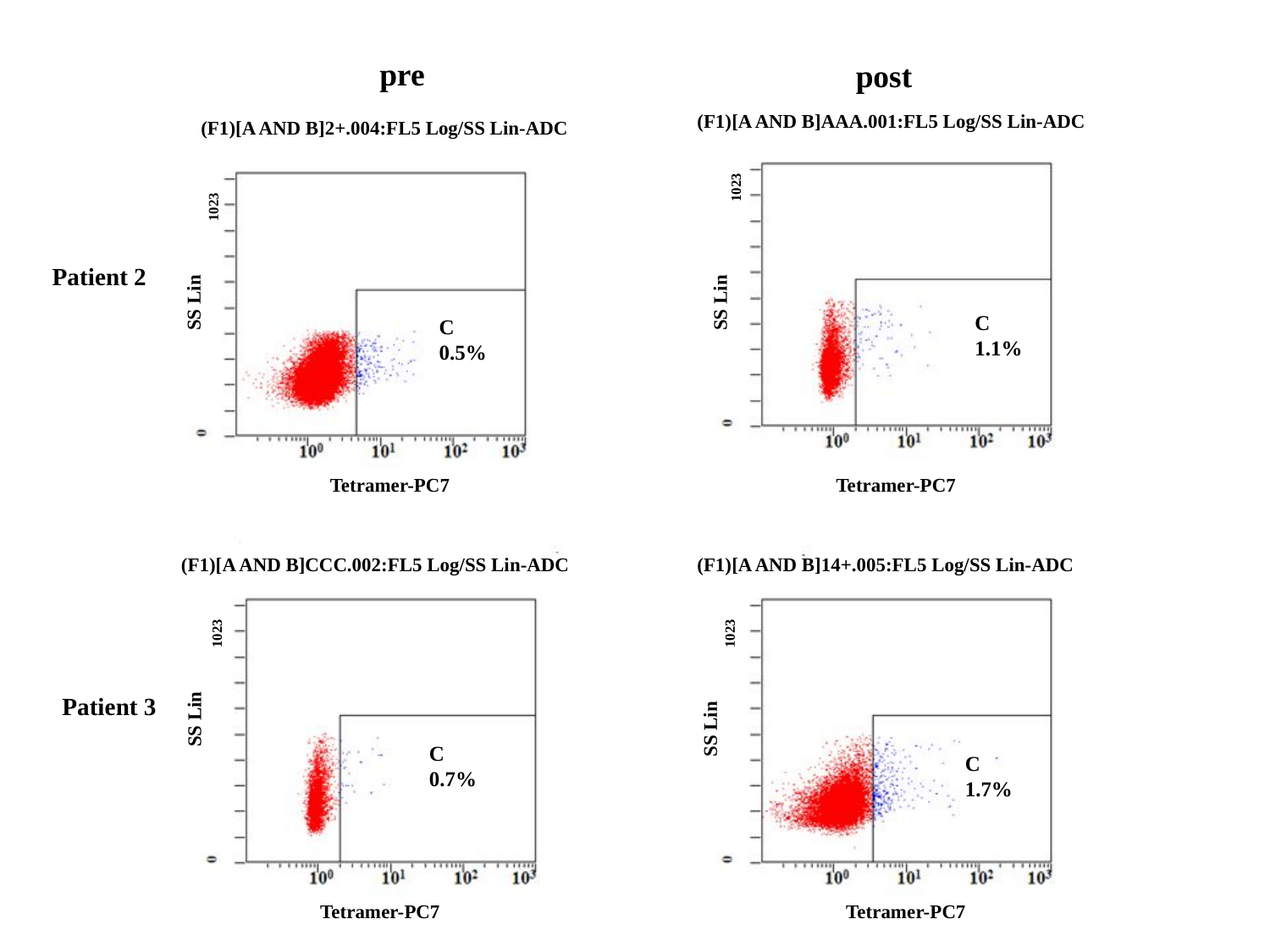

pre
post
(F1)[A AND B]AAA.001:FL5 Log/SS Lin-ADC
(F1)[A AND B]2+.004:FL5 Log/SS Lin-ADC
1023
1023
SS Lin
SS Lin
Patient 2
C
1.1%
C
0.5%
Tetramer-PC7
Tetramer-PC7
(F1)[A AND B]CCC.002:FL5 Log/SS Lin-ADC
(F1)[A AND B]14+.005:FL5 Log/SS Lin-ADC
1023
1023
SS Lin
SS Lin
Patient 3
C
0.7%
C
1.7%
Tetramer-PC7
Tetramer-PC7
